# A Cross-Sectional Study of Physical Fitness and Risk of Hypertension in Korean Adults Aged 20–59

**DOI:** 10.3390/healthcare11142057

**Published:** 2023-07-18

**Authors:** Bogja Jeoung, Jiyoun Kim

**Affiliations:** Department of Exercise Rehabilitation, Gachon University, 191 Hambakmoe-ro, Yeonsu-gu, Incheon 21936, Republic of Korea; bogja05@gachon.ac.kr

**Keywords:** hypertension, Korean adults, physical fitness level, national fitness award project, risk factor

## Abstract

Intervention for hypertension in young age groups is very important. Adults in their 30s and 40s in Korea are the main producers of economic activity. Stress in work life, frequent drinking and smoking, an unhealthy diet, and a lack of physical activity are the biggest factors that increase the risk of high blood pressure. This study analyzed obesity-related body composition, physical fitness, and risk according to age and sex by analyzing population-based hypertension and physical fitness trends in individuals aged 20–59 years in 360,321 Korean adults via National Physical Award Project (NFAP) data points collected from 2012 to 2019. The functional fitness test battery for adults was composed of seven components: (a) aerobic endurance (2-min step), (b) upper body muscle strength (hand grip strength), (c) lower body muscle endurance (sit-ups), (d) flexibility (sit and reach), (e) cardiopulmonary endurance (progressive aerobic capacity endurance run), (f) body compositions (body mass index [BMI] and waist circumference [WC]), and (g) blood pressure. For all items, there was a significant difference in blood pressure according to people’s physical fitness levels. Specifically, for obesity-related BMI and WC, the higher the obesity, the higher the blood pressure (post hoc, obesity > overweight > normal > underweight). It was also confirmed that the lower the grade, that is, the weaker the grip, the higher the blood pressure (post hoc, 4 > 3 > 2 > 1). Subsequently, in identifying the risk factors for high blood pressure, the physical fitness level (Model 1) and obesity-related indicator (Model 2) differed by 1.024 and 1.335 times, respectively. Finally, it was confirmed that the risk of high blood pressure in the age and gender model (Model 3) increased by 1.388 times. In addition to the recommendation for changes in blood pressure, significant differences in blood pressure according to physical fitness and significant effects on blood pressure risk in terms of age, obesity-related body composition, and physical fitness were confirmed.

## 1. Introduction

The Korean Ministry of Health and Welfare established the Health Plan 2030 to prevent an increasing number of chronic diseases in an aging population. It consists of promoting health policies and related projects to lower the prevalence of high blood pressure, diabetes, and dyslipidemia, which are the leading stages of chronic diseases (cardiovascular disease [CVD], diabetes, chronic respiratory disease, and cancer) with high medical expenses [1]. High blood pressure is one of the most common medical conditions related to CVD and is a major risk factor for global mortality [2]. The prevalence of hypertension in Korea is reported to be 11.3%, 19.2%, 32.1%, 46.9%, and 64.7% for those aged 30–39, 40–49, 50–59, 60–69, and ≥70, respectively [3]; it is defined as a very high disease in 34.2% of adults over the age of 30 years [4]. The estimated cost of treating hypertension is KRW 3830 billion, accounting for 4% and 16% of the total medical expenses for the country and chronic diseases, respectively [5]. Specifically, as the onset of hypertension at a young age is reported to be closely related to the onset of CVD and the shortening of lifespan [6], intervention for hypertension in the young age group is very important. Men in their 30s and 40s in Korea are the main producers of economic activity. Stress in work life, frequent drinking and smoking, an unhealthy diet [7], and a lack of physical activity are the biggest factors that increase the risk of high blood pressure [8].

Studies have suggested that regular physical activity has important health benefits for chronic diseases and high blood pressure [9,10,11]. Regular physical activity is also an important variable but the level of physical fitness itself is closely related to CVDs, including high blood pressure. Apart from physical activity, it was argued that physical fitness level should be classified as an independent risk factor [12]. Moreover, since the participants were limited to older adults, more research efforts are needed to identify various factors in adulthood for health care and intervention.

Physical fitness is reported to be closely related to health improvements and a reduction in medical expenses [13]. Recent studies conducted in Korea examined the relationship between physical fitness and medical expenses [14]. Lee et al. [14] conducted a study within the National Fitness Award Project (NFAP) which provides physical evaluations and exercise prescriptions through physical fitness measurements. The study compared the annual medical expenses of non-participating groups with similar income and demographic characteristics, revealing that the expenses were $200 higher. Hence, physical fitness serves as an important indicator for maintaining and improving health. However, considering the influence of different biological and environmental factors such as gender, age, and race, obtaining the latest normative reflectance values from large-scale samples at the national level is essential.

The NFAP is currently being conducted in Korea as a state-led public sports welfare service that measures and evaluates physical fitness through scientific methods to improve participants’ physical fitness and health. As of 2021, more than 70 fitness certification centers nationwide have conducted physical fitness measurements to determine grades for each factor according to age group standards and provide information on current physical fitness and motivation for physical activities [15]. This study’s purpose was to establish a four-grade system for physical fitness levels in Koreans using NFAP data [16]. Additionally, it aimed to examine the association between physical fitness and blood pressure as well as identify the risk of high blood pressure in relation to preventive physical fitness levels in Korea.

Therefore, this study analyzes obesity-related body composition, physical fitness, and risk according to age and sex by analyzing population-based hypertension and physical fitness trends in individuals aged 20–59 years in Korea using 100 national physical fitness data points collected from 2012 to 2019. The results of such a study recognize the importance of physical fitness in reducing the possibility of high blood pressure and are used as evidence to suggest mediating measures for high blood pressure in preparation for an aging population.

## 2. Materials and Methods

### 2.1. Research Data

The data used in this cross-sectional study were drawn from the Korea Institute of Sport Science Fitness Standards as part of the NFAP which is open to the public. The Korea Sports Promotion Foundation released eight years of data (2012–2019) during data collection. Nationally, there are 81 test centers across 17 regions in Korea. The participants were aged 20–59. Functional fitness test data from 360,321 individuals (159,201 males [44.18%] and 201,120 females [55.82%]) who voluntarily participated in the centers from 2012 to 2019, along with their demographic information, were used in this study. According to the 2018 Korean Society of Hypertension Guidelines, the standard for hypertension is defined as normal for <120 mmHg, borderline hypertension for 120–139 mmHg, and hypertension for >140 mmHg [17]. Table 1 shows the status of the data collected according to the hypertension threshold.

### 2.2. Functional Fitness Measurement

The functional fitness test battery for adults was composed of seven components: (a) aerobic endurance (2-min step), (b) upper body muscle strength (hand grip strength), (c) lower body muscle endurance (sit-ups), (d) flexibility (sit and reach), (e) cardiopulmonary endurance (progressive aerobic capacity endurance run [PACER]), (f) body compositions (body mass index [BMI] and waist circumference [WC]), and (g) blood pressure. All functional fitness parameters were measured voluntarily at the designated centers. The validity of the individual tests was not examined because this study used publicly released data from the NFAP which were developed through rigorous validation methods and successfully implemented and utilized for national fitness tests [18]. All test procedures were performed in accordance with the relevant guidelines and regulations and were facilitated by certified national professional health and fitness instructors.

The measured values for each item, such as strength, muscle endurance, flexibility, and cardiopulmonary endurance, were used, as were the criteria for each gender and age. As for the grades, the following criteria apply: grade 1: ≥70%, grade 2: ≥50%, grade 3: ≥30%, and grade 4: <30%. As for the percentile standard, the five-year interval (20–25, 25–29, 30–34, 35–39, 40–44, 45–49, 50–54, and 55–59 years old) was applied (Table 2) [16].

#### 2.2.1. Muscular Strength (Grip Strength)

The participants were instructed to hold a hand gripper with the second knuckle of their hand, stretch their arms forward, keep their body and arm distanced by 15°, and grip it as hard as they could for five seconds with their posture unchanged. The left and right arms were measured twice, one after another, and the best score for each arm was recorded in 0.1 kg units. The relative handgrip strength (handgrip strength ÷ body weight × 100) was then calculated [19].

#### 2.2.2. Muscular Endurance (Sit-Ups)

Sit-ups were used to evaluate muscular endurance, which is the ability to continuously utilize muscles for the same movement. The number of sit-ups per minute was counted. The participants were asked to lie on a rubber mat and ensure that their backs were flat. They were instructed to keep their hands behind their heads and flex their knees at an angle of 90° while keeping their legs apart. The assistant then held each participant’s ankles and the individual had to raise their upper body and touch their elbows to their knees. This action was counted as one sit-up and was repeated continuously for the specified period of time [20].

#### 2.2.3. Flexibility (Sit-and-Reach)

The participants were instructed to remove their shoes and sit with their knees stretched forward until their soles were flat against the measurement device. The participants were then told to keep their knees pressed flat on the floor with palms touching the box facing downward, bend their upper bodies, and extend them as far forward as possible. The measurements were performed twice and the best record was selected and recorded in units of 0.1 cm [19].

#### 2.2.4. Cardiopulmonary Endurance (PACER)

The participants were familiarized with and followed the standard PACER procedure under the supervision of trained research personnel. Participants were instructed to run back and forth across a marked 20-m course in a straight line, pivot, turn around, complete a lap, and pace themselves in accordance with an audio recording. The participants were instructed to continue running until their pace could no longer be maintained. Strong verbal encouragement was provided by research personnel to continue running for as long as possible. The participants completed the PACER test individually. The total number of laps completed during the test was also recorded [21].

#### 2.2.5. Body Compositions (BMI and WC)

The BMI was calculated by dividing body weight (kg) by height in square meters (m^2^). The percentage of body fat was measured using bioelectrical impedance analysis with a bioelectrical impedance analysis device (InBody 720, BioSpace, Seoul, Republic of Korea). The BMI was classified into underweight (<18.5 kg/m^2^), normal weight (18.5–24.9 kg/m^2^), overweight (25–29.9 kg/m^2^), and obese (≥30 kg/m^2^) [22]. WC was measured around the abdomen at the umbilicus to the nearest 0.1 cm using a flexible metric measuring tape with the participants in a standing position and divided into four stages: male: very low <80 cm, low 80–99 cm, high 100–120 cm, and very high >120 cm and female: very low <70 cm, low 70–89 cm, high 90–109 cm, and very high >110 cm [23].

### 2.3. Blood Pressure

Blood pressure was measured once using an electronic blood pressure meter (BPBIO330, BioSpace, Seoul, Republic of Korea) after stabilization for at least 5 min while sitting comfortably in a chair in a quiet environment. Precautions were taken during the measurement process to account for factors such as smoking, alcohol, and caffeine intake within 30 min of measurement [24].

### 2.4. Statistical Analyses

The SPSS 23.0 Version (IBM Co., Armonk, NY, USA) was used for data analysis. The chi-square test and one-way analysis of variance were performed to compare the frequency and average values based on the participants’ general characteristics and blood pressure according to physical fitness; if there was a significant difference, Scheffé’s post hoc test was performed. A logistic regression analysis was performed to determine whether the participants’ physical fitness affected their blood pressure. Furthermore, the results were analyzed by performing a multi-logistic regression analysis by applying the following sequential models to identify the day of the effect on blood pressure: Model 1 was conducted by additionally correcting for physical fitness level, Model 2 for obesity-related body composition, and Model 3 for age and sex. For all tests, the significance level was set at *p* < 0.05.

## 3. Results

### 3.1. NFAP from 2012 to 2019 According to Hypertension Standards

Table 1 shows the current status of the collected data according to the hypertension standards. Of the 360,321 adults aged 20–59 who were analyzed, 189,938 (51.0%) and 44,683 (12.4%) were confirmed to have borderline hypertension (pre-hypertension) and hypertension, respectively. The incidence of borderline hypertension or hypertension was higher in men than in women and the rate of hypertension gradually increased with the measurement period.

### 3.2. Difference in Blood Pressure by Fitness Level

Table 3 shows the results of confirming the differences in blood pressure according to physical fitness level. Physical fitness was confirmed for four types: muscle strength, muscle endurance, flexibility, and cardiopulmonary endurance as well as obesity-related body composition (BMI and WC). For all items, there was a significant difference in blood pressure according to their physical fitness level (*p* < 0.001). Specifically, in obesity-related BMI and WC, the higher the obesity, the higher the blood pressure (post hoc, obesity > overweight > normal > underweight; *p <* 0.001). It was also confirmed that the lower the grade, that is, the weaker the grip, the higher the blood pressure (post hoc, 4 > 3 > 2 > 1; *p* < 0.001). In sit-ups and PACER, it was confirmed that the lower the grade, the lower the blood pressure (*p <* 0.001). However, in the sit-and-reach test, it was confirmed that blood pressure was low at grade 4 (post hoc, 3, 2, 1 > 4; *p* < 0.001).

### 3.3. Odds Ratio (95% Confidence Interval [CI]) for Blood Pressure Stage and Fitness Level Baseline Variables

Based on the odds ratios (OR) for blood pressure stage and physical fitness level, the risks of borderline hypertension and hypertension were confirmed as follows (Table 4): BMI increased 3.22 times (OR 3.22, [95% CI 3.07–3.37]; *p* < 0.001) in the obese group compared to the normal group with borderline hypertension and 9.71 times (OR 9.71, [95% CI 9.71–10.23]; *p* < 0.001) in the obese group with high blood pressure. WC also increased the risk by 2.78 times (OR 2.78, [95% CI 2.26–3.41]; *p* < 0.001) in the obese group compared to the normal group with borderline hypertension and 6.20 times (OR 6.20, [95% CI 4.96–7.75]; *p* < 0.001) in the obese group with high blood pressure. In terms of physical fitness, Griff’s stranger also increased the risk by 1.07 times (OR 1.07, [95% CI 1.05–1.09]; *p* < 0.001) in grade 4 (OR 1.43, [95% CI 1.38–1.47]; *p* < 0.001) in high blood pressure compared to grade 1. Sit-ups, a muscular endurance test, showed a decrease in risk in grades 2 (OR 0.98) and 3 (OR 0.94) compared to grade 1, with the highest physical fitness level in borderline hypertension. However, in hypertension, the risk increased 1.06 times in grade 4 (OR 1.06 [95% CI 1.03–1.09]; *p* < 0.001).

Sit-and-reach, a flexibility indicator, showed no significant difference in grade 2 compared to grade 1 with the highest physical fitness level in both borderline and hypertension and the risk was rather reduced in grade 4 (OR 0.96, [95% CI 0.94–0.95]; *p* < 0.001; OR 0.97, [95% CI 0.94–0.95]; *p* = 0.016). Finally, PACER, an indicator of cardiopulmonary endurance, confirmed that the risk increased in grade 41 (OR 1.11, [95% CI 1.07–1.15]; *p* < 0.001) compared to grade in hypertension.

### 3.4. Odds Ratios with a 95% Confidence Interval for the National Fitness Award Project Data on Hypertension Risk

To identify the risk factors for hypertension, Models 1, 2, and 3 were corrected for the following parameters: fitness level, obesity index (BMI and WC), and age and sex (Table 5). Compared to normal blood pressure, an increase in blood pressure owing to borderline hypertension and hypertension is a factor that shows a significant risk depending on a person’s physical fitness level, obesity, age, and sex. Specifically, the physical fitness level differed by 1.015 times (OR 1.015, [95% CI 1.013–1.016]; *p* < 0.001), hypertension by 1.02 times (OR 1.02, [95% CI 1.02–1.26]; *p* < 0.001), obesity-related indicators by 1.163 times (OR 1.163, [95% CI 1.158–1.168]; *p* < 0.001), and hypertension by 1.336 times.

Finally, it was confirmed that the risk of differences in age and sex in Model 3 increased 1.194 times in borderline hypertension (OR 1.194, [95% CI 1.189–1.198]; *p* < 0.001) compared to 1.388 times in high blood pressure (OR 1.388, [95% CI 1.381–1.389]; *p* < 0.001).

## 4. Discussion

Many studies are attempting diverse research with an interest in exercise to prevent high blood pressure. However, after 2015, most of these studies were limited to the analysis of the effects of exercise on a drop in blood pressure and the analysis of physical fitness factors in older adults [6,24,25]. This study went one step further from research that emphasized the importance of regular exercise to examine whether a person’s physical fitness level is also an important independent risk factor in preventing high blood pressure: this study identified risk factors able to prevent high blood pressure. High blood pressure mainly occurs in daily life owing to a lack of physical activity and poor eating habits [26]. This is closely related to the causes of diseases such as obesity. In this study, a significant difference was observed between blood pressure according to BMI and WC grades, which are obesity-related body compositions. According to a study of more than one million male and female participants in the United State, conducted by the Community Hypertension Evaluation Clinic, the prevalence of hypertension in overweight participants aged 20–39 was twice that in normal-weight participants and three times that in underweight participants [27]. According to the Framingham Heart Study, obesity is the cause of 78% of primary hypertension in men and 65% in women [28]. Additionally, a study that analyzed the risk factors for high blood pressure in 1269 middle-aged and older participants in Korea reported that the risk of developing high blood pressure was 1.58 times higher than that of normal weight in BMI 25–29.9 and 1.82 times higher than that of normal weight in BMI 30 [29]. Furthermore, according to a follow-up study, WC prediction ability together with BMI indicates the risk of developing hypertension and supports similar results to this study. Given the strong correlation between obesity and high blood pressure, the prevention and management of obesity are very important to control the prevalence of hypertension as the Korea Centers for Disease Control and Prevention reported a 34.2% increase in the prevalence of hypertension by 2021. Since most of the preceding studies evaluated middle-and old-age groups of ≥50 years [30], the results of this study are interpreted as important data for controlling blood pressure prevention purposes.

Research on medical expenses according to the level of physical fitness has been continuously conducted and it has been reported that a high level of physical fitness is closely related to decreased medical expenses [31]. In this study, high blood pressure was confirmed at a low fitness level for all physical fitness indicators except flexibility. Loss of muscle mass and muscle strength occurs continuously in adulthood, accelerating from middle to old age [32,33]. Muscle loss limits daily life in old age and increases the mortality rate of sarcopenia and CVDs [34]. Grip strength is used as a muscle strength measurement item in clinical and epidemiological studies because it can measure muscle strength simply and non-invasively and is not only an indicator of muscle strength but also an important biomarker of health and disease [35]. Studies have reported an association between lower grip strength in middle-aged and older adults and a higher prevalence of chronic diseases, regardless of the specific type of chronic disease [36]. Grip strength is reported to peak during the 30s [37] and then gradually decrease after the age of 50 [38]. Several studies related to high blood pressure and muscle function have identified low grip levels as well as an increase in the prevalence of hypertension and defined them as important means of identifying the association between grip and CVD [39]. In contrast, high grip levels after middle age are not only closely related to a decrease in the prevalence of diseases but are also known to play a positive role in reducing the risk of early death from chronic diseases [40,41].

This study’s results confirmed that individuals with high blood pressure have a 1.43 times higher risk of having lower grip strength compared to those in grade 1 of grip level. This result is interpreted in a similar context to studies that reported that maintaining a high grip level can induce a positive effect.

The sit-ups test represents lower extremity muscle strength and is also used as an indicator of muscle loss risk in old age [42]. In this study, grade 4 was 1.06 times higher in the high blood pressure group than in grade 1; the higher the muscle strength of the upper and lower body of the middle-aged and older adults exposed to high blood pressure, the lower the risk of death [43,44]. Decreased muscle strength is associated with increased oxidative stress [45] and chronically induced inflammatory molecules [46] as well as mitochondrial biosynthesis and function [47], suggesting that physical fitness, through regular physical activity, reduces vascular resistance, increases vascular expansion, and can play a positive role in the main mechanisms of high blood pressure, such as blood circulation related to capillary transport owing to increased albumin vascular inflow [48,49,50].

Meanwhile, studies have reported a correlation between a high level of flexibility, a reduced risk of arteriosclerosis [51], and high blood pressure [52], regardless of other confounding factors. In a Japanese study involving 102,948 individuals aged 29–92, Gondo et al. [52] identified a common point between age-related changes, determinants of flexibility, and blood pressure. Collagen fibers are greatly involved in the rigidity of ligaments, joint capsules, and fascia, which are structural factors related to flexibility, and such fibers are also greatly involved in arterial wall rigidity in blood pressure regulation. Particularly notably is the accelerated decline in trunk flexibility that occurs after middle age, highlighting the importance of examining trends in adulthood. However, contrary to expectations, this study revealed a lower risk of hypertension in individuals with lower flexibility grades. Similarly, Yamada et al. [53] found that flexibility did not have a significant positive impact on the arteriosclerosis index or blood pressure. Nevertheless, it is evident that exercise effectively controls high blood pressure in hypertensive patients.

Finally, the risk of hypertension was confirmed by dividing the 100 national physical fitness datasets into Model 1, corrected for muscle strength, muscle endurance, flexibility, and cardiopulmonary endurance; Model 2, corrected for obesity-related body composition of BMI and WC; and Model 3, corrected for sex and age. The risk of hypertension was the highest in Model 3 for sex and age which can be interpreted in the same sense as the high incidence of hypertension [3] in older adults, as published in previous studies or statistical data. Subsequently, the risk was confirmed in terms of obesity-related body composition and physical fitness. As shown in the association of the risk of high blood pressure with obesity level in older adults, the same trend of high blood pressure risk was confirmed in adults in this study [29,30]. Additionally, it is important to pay attention to the significant differences in the risk of high blood pressure according to the level of physical fitness; it is believed that participation in physical activities [9,10] that help maintain and improve physical fitness should be supported.

This study has several advantages. First, the sample size was large (360,321 participants over eight years); the data were collected from physical fitness data for adults aged 20–59 in Korea and measured by a specialized institution called the NFAP. Therefore, the statistical analysis was performed using a significantly extensive dataset. Consequently, the ample sample size increases the likelihood of observing statistical significance, even for minor differences in the result values. Second, this was the only study to confirm and analyze the risk of physical fitness and blood pressure in domestic adults. However, a limitation of the study is the fact that the direct explanation of the factors contributing to the physical fitness level was challenging owing to insufficient data on health-related surveys and lifestyle factors (such as exercise participation experience). This lack of information not only affected the physical fitness indicators but also other crucial factors. However, if these points are included in future studies, they can be used as a new independent variable that can control blood pressure as well as a disturbance factor in physical fitness. Therefore, future studies will require more detailed and well-structured follow-up investigations, encompassing appropriate sample sizes and providing comprehensive insights into specific disease types, age groups, racial differences, gender disparities, and other relevant factors. Furthermore, considering that one of the goals of chronic disease management at the national level is to control blood pressure, prevent CVD caused by a rise in blood pressure, and reduce mortality, it is important to identify related factors, including not only those affected by high blood pressure but also those at all stages of high blood pressure [54]. The results also indicate that the physical fitness data of the NFAP can play an important role as an indicator for identifying and preventing risk factors for blood pressure and represent domestic health indicators. Moreover, as a preventive measure, it is necessary to check physical fitness indicators and prescribe and manage physical activity accordingly.

This study was limited by its retrospective cohort design which prevented the evaluation of the cause and effect between physical fitness level and blood pressure; only the correlation between these factors was assessed. Future studies will require a more detailed experimental design to investigate the cause of the correlation. Moreover, the participant age range included in this study is large and may have affected the results as the cause of associated factors may be very different in participants of different ages.

## 5. Conclusions

This study investigated the relationship between blood pressure and physical fitness in adults aged 20–59 based on the NFAP. In addition to the recommendation for changes in blood pressure, significant differences in blood pressure according to physical fitness were confirmed and significant effects on blood pressure risk were confirmed in terms of age, obesity-related body composition, and physical fitness. However, as this was a cross-sectional study centered on physical fitness indicators, accompanying diseases or exercise histories could not be confirmed. Most studies related to blood pressure and physical fitness were conducted on older adults; data on adults aged 20–59 using both blood pressure and physical fitness indicators are insufficient. Therefore, despite the limitations of the data surveys, it is expected to be a meaningful research report in that it directly checks the risk indicators of blood pressure and conducts research at a preventative level. In future studies, systematic epidemiological studies are required to investigate the relationship between blood pressure and physical fitness.

## Figures and Tables

**Table 1:** The National Fitness Award Project from 2012 to 2019 according to hypertension standards.

Characteristics		Total n (%)	Nomal (<120 mmHg) n (%)	Borderline Hypertension (121–139 mmHg) n (%)	Hypertension(>140 mmHg) n (%)	F	*p*
Variables	Category						
Age	20–29	130,790 (100)	48,790 (37.3)	68,388 (52.3)	13,612 (10.4)	649.1	0.001 ***
	30–39	33,773 (100)	12,516 (37.1)	17,027 (50.4)	4230 (12.5)		
	40–49	90,292 (100)	33,626 (37.2)	45,534 (50.4)	11,132 (12.3)		
	50–59	105,466 (100)	36,768 (34.9)	52,989 (50.2)	15,709 (12.3)		
Sex	Male	159,201 (100)	35,069 (22.0)	93,650 (58.8)	30,482 (19.1)	17,892.1	0.001 ***
	Female	201,120 (100)	96,631 (48.0)	90,288 (44.9)	14,201 (7.1)		
Survey year	2012	8779 (100)	4170 (47.5)	4078 (46.5)	531 (6.0)	896.7	0.001 ***
	2013	25,171 (100)	10,920 (43.4)	12,293 (48.8)	1958 (7.8)		
	2014 2015	39,175 (100)	15,378 (39.3)	19,674 (50.2)	4123 (10.5)		
		51,047 (100)	19,078 (37.4)	25,725 (50.4)	6244 (12.2)		
	2016	55,520 (100)	19,499 (35.1)	28,062 (50.5)	7959 (14.3)		
	2017	63,604 (100)	21,729 (34.2)	33,808 (53.2)	8067 (12.7)		
	2018 2019	78,212 (100)	27,892 (35.7)	40,325 (51.6)	9995 (12.8)		
		38,813 (100)	13,034 (33.6)	19,973 (51.5)	5806 (15.0)		
Total		360,321 (100)	131,700 (36.3)	183,938 (51.0)	44,683 (12.4)		

Data are presented as n (%). *** *p* < 0.001; tested by performing the chi-square.

**Table 2:** Guidelines for physical fitness grades in the National Fitness Award Project.

Grade	Age (Years)	Male						Female					
		Grip Strength (%)	Sit-Ups (Reps)	Sit-and-Reach (cm)	PACER(Reps)	WC (cm)	BMI (kg/m^2^)	Grip Strength(%)	Sit-Ups (Reps)	Sit-and-Reach (cm)	PACER(reps)	WC (cm)	BMI (kg/m^2^)
1	19–24	62.6	55	16.1	62	<80	<18.5	46.8	36	19.7	30	<70	<18.5
	25–29	62.4	51	14.9	54			47.0	33	12.7	28		
	30–34	62.6	47	14.2	49			49.6	31	12.9	26		
	35–39	62.1	45	14.0	45			47.5	31	13.0	25		
	40–44	62.8	44	14.2	42			47.1	30	13.1	24		
	45–49	62.0	41	13.6	40			46.2	28	13.4	23		
	50–54	60.5	38	13.9	35			44.7	24	13.8	21		
	55–60	59.4	35	13.3	31			43.2	20	14.5	18		
2	19–24	57.2	48	11.1	52	80–99	18.5–24.9	42.4	30	14.9	25	70–89	18.5–24.9
	25–29	57.0	45	10.1	44			42.6	27	13.8	23		
	30–34	57.2	41	9.4	40			45.2	25	13.8	21		
	35–39	56.6	39	9.3	37			43.1	25	13.9	20		
	40–44	57.2	38	9.5	35			42.8	25	14.0	20		
	45–49	56.7	36	9.1	32			41.9	22	14.4	18		
	50–54	55.3	32	9.3	28			40.6	19	14.9	16		
	55–60	54.2	29	8.6	25			39.2	15	15.8	14		
3	19–24	51.8	42	6.1	41	100 –120	25–29.9	38.0	23	10.1	19	90–109	25–29.9
	25–29	51.6	38	5.3	34			38.2	21	9.1	17		
	30–34	51.8	35	4.6	31			40.8	19	9.4	16		
	35–39	51.1	33	4.6	28			38.7	19	10.1	15		
	40–44	51.6	32	4.8	27			38.5	19	10.4	15		
	45–49	51.4	30	4.6	24			37.6	16	10.7	14		
	50–54	50.1	26	4.7	21			36.5	13	11.7	12		
	55–60	49.0	23	3.9	18			35.2	9	11.9	11		
4	3rd grade certification criteria and below					>120	>30	3rd grade certification criteria and below				>110	>30

PACER, progressive aerobic capacity endurance run; WC, waist circumference; BMI, body mass index.

**Table 3:** Difference in blood pressure by fitness level.

Variable	Fitness Level	Blood Pressure (Systolic)		F	*p*	Post Hoc
		Numbers	Mean ± Standard Deviation			
Grip strength	1	147,008	122.95 ± 12.98	233.126	<0.001 ***	4 > 3 > 2 > 1
	2	76,233	123.66 ± 13.27			
	3	64,516	123.94 ± 13.45			
	4	72,564	124.45 ± 13.77			
Sit-ups	1	135,363	123.70 ± 13.10	42.062	<0.001 ***	4 > 3, 2 > 1
	2	71,275	123.50 ± 13.16			
	3	72,786	123.32 ± 13.31			
	4	80,624	124.04 ± 13.35			
Sit-and-reach	1	108,162	123.65 ± 13.17	11.744	<0.001 ***	3, 2, 1 > 4
	2	75,236	123.64 ± 13.25			
	3	69,119	123.76 ± 13.25			
	4	107,804	123.35 ± 13.49			
PACER	1	74,370	123.32 ± 12.65	12.401	<0.001 ***	3, 4 > 1
	2	90,286	123.52 ± 13.44			
	3	100,959	123.71 ± 13.37			
	4	94,706	123.67 ± 13.57			
BMI	Underweight	11,213	115.29 ± 11.62	9866.057	<0.001 ***	4 > 3 > 2 > 1
	Normal	235,384	121.55 ± 12.74			
	Overweight	98,344	128.01 ± 12.91			
	Obesity	15,380	132.33 ± 13.34			
WC	Very low	51,333	121.72 ± 12.70	1252.488	<0.001 ***	4 > 3 > 2 > 1
	Low	286,607	123.55 ± 13.32			
	High	21,589	128.00 ± 13.20			
	Very high	792	132.19 ± 13.33			

*** *p* < 0.001; tested by performing one-way analysis of variance. Post hoc, Scheffé; PACER, progressive aerobic capacity endurance run; BMI, body mass index; WC, waist circumference.

**Table 4:** Odds ratio (95% confidence interval) for blood pressure stage and fitness level baseline variables.

Characteristics		Borderline Hypertension (121–139 mmHg)		Hypertension (>140 mmHg)	
Variables category		Odds ratio (95% confidence interval) and *p*			
Grip strength	1st	Reference		Reference	
	2nd	1.05 (1.03–1.07)	<0.001 ***	1.19 (1.15–1.22)	<0.001 ***
	3rd	1.07 (1.05–1.09)	<0.001 ***	1.27 (1.23–1.31)	<0.001 ***
	4th	1.07 (1.05–1.09)	<0.001 ***	1.43 (1.38–1.47)	<0.001 ***
Sit-ups	1st	Reference		Reference	
	2nd	0.98 (0.96–1.00)	0.049 *	0.95 (0.93–0.98)	<0.001 ***
	3rd	0.94 (0.93–0.96)	<0.001 ***	0.93 (0.90–0.96)	0.010 **
	4th	1.00 (0.98–1.02)	0.574	1.06 (1.03–1.09)	<0.001 ***
Sit-and-reach	1st	Reference		Reference	
	2nd	1.01 (0.98–1.03)	0.579	1.01 (0.97–1.04)	0.655
	3rd	1.04 (1.01–1.06)	0.002 **	1.02 (0.99–1.05)	0.189
	4th	0.96 (0.94–0.98)	<0.001 ***	0.97 (0.94–0.99)	0.016 *
PACER	1st	Reference		Reference	
	2nd	0.91 (0.89–0.93)	<0.001 ***	1.10 (1.06–1.13)	<0.001 ***
	3rd	0.95 (0.93–0.97)	<0.001 ***	1.14 (1.11–1.18)	<0.001 ***
	4th	0.93 (0.91–0.95)	<0.001 ***	1.11 (1.07–1.15)	<0.001 ***
BMI	Normal	Reference		Reference	
	Underweight	0.47 (0.45–0.49)	<0.001 ***	0.20 (0.17–0.22)	<0.001 ***
	Overweight	2.10 (2.06–2.14)	<0.001 ***	3.90 (3.81–4.00)	<0.001 ***
	Obesity	3.22 (3.07–3.37)	<0.001 ***	9.71 (9.21–10.23)	<0.001 ***
WC	Low	Reference		Reference	
	Very low	0.89 (0.87–−0.91)	<0.001 ***	0.62 (0.60–0.64)	<0.001 ***
	High	1.64 (1.59–1.70)	<0.001 ***	2.38 (2.28–2.48)	<0.001 ***
	Very high	2.78 (2.26–3.41)	<0.001 ***	6.20 (4.96–7.75)	<0.001 ***

* *p* < 0.05, ** *p* < 0.01, *** *p* < 0.001; tested by performing logistic regression analysis; PACER, progressive aerobic capacity endurance run; BMI, body mass index; WC, waist circumference.

**Table 5:** Odds ratios with 95% confidence intervals for the National Fitness Award Project data on hypertension risk.

Characteristics		(a) Model 1		(b) Model 2		(c) Model 3	
Variable		OR (95% CI)	*p*	OR (95% CI)	*p*	OR (95% CI)	*p*
Hypertention	≤120 (ref)						
	121–139	1.015 (1.013–1.016)	<0.001 ***	1.163 (1.158–1.168)	<0.001 ***	1.194 (1.189–1.198)	<0.001 ***
	>140	1.024 (1.022–1.260)	<0.001 ***	1.335 (1.326–1.343)	<0.001 ***	1.388 (1.381–1.389)	<0.001 ***

*** *p* < 0.001; tested by performing multiple logistic regression analysis. (a) Model 1: further adjusted for fitness level (total fitness test score), (b) Model 2: further adjusted for obesity factor (body mass index and waist circumference), (c) Model 3: further adjusted for age and sex; OR: odds ratio; CI, confidence interval; PACER, progressive aerobic capacity endurance run; BMI, body mass index; WC, waist circumference.

## Data Availability

The datasets used and/or analyzed during the current study are available from the corresponding author upon reasonable request.
